# Toward Better Clinical Governance: A Six Sigma Analysis of Patient Satisfaction Determinants in a Tertiary Care Government Hospital

**DOI:** 10.7759/cureus.94068

**Published:** 2025-10-07

**Authors:** Suyash Singh, Purushottam Kumar, Abhay Singh, Samir Shukla, Kurvatteppa Halemani, Pankaj Kumar

**Affiliations:** 1 Department of Neurosurgery, AIIMS (All India Institute of Medical Sciences) Raebareli, Raebareli, IND; 2 Department of Community and Family Medicine, AIIMS (All India Institute of Medical Sciences) Raebareli, Raebareli, IND; 3 Department of Indian Audit and Accounts, Government of India, Allahabad, IND; 4 Department of Nursing, AIIMS (All India Institute of Medical Sciences) Raebareli, Raebareli, IND

**Keywords:** clinical governance, health services, patient satisfaction, quality management, service quality, six sigma

## Abstract

Background: The quality of hospital service and clinical governance depends heavily on patient satisfaction as an essential indicator. The identification of satisfaction determinants helps healthcare providers enhance their delivery methods, particularly in public hospitals with limited resources.

Objective: The research investigated patient satisfaction while applying Six Sigma principles to determine its essential determinants in a tertiary care government hospital.

Methods: A facility-based cross-sectional study took place from November 2023 to December 2024 by selecting 400 patients through random methods. The 19-item structured questionnaire assessed patient satisfaction in three domains that included service utilization, patient-provider interaction, and facility-related factors. A five-point Likert scale evaluated the general satisfaction of patients. The researchers used SPSS to analyze the data through factor analysis and bivariate regression to identify the predictors.

Results: The patient satisfaction level reached 80.4% in the study. The clinical care delivered by doctors and nursing staff proved to be the primary factor that satisfied patients. Facility-related amenities showed substantial gaps because patients expressed dissatisfaction with toilet cleanliness, waiting space availability, dietary services, and pharmacy queue lengths. The unclean state of toilets proved to be the primary cause of patient dissatisfaction because 40% of participants expressed their discontent. The study revealed that elderly patients together with those who reported poor health status demonstrated lower satisfaction levels. The analysis showed that service quality improvements directly resulted in increased patient satisfaction.

Conclusion: The overall satisfaction scores were positive, but nonclinical service domains require substantial improvement. The satisfaction levels could increase substantially through hospital hygiene improvements, outpatient procedure optimization, patient-provider communication enhancement, and technology-based queue management systems. The implementation of the Six Sigma methodology for regular monitoring enabled healthcare providers to develop evidence-based strategies that enhance clinical governance in government hospitals.

## Introduction

The large number of patient footfalls in Government Hospitals in any developing country is one of the most challenging areas for quality and safety improvement [1,2]. Although automation and information technology have eased most crowd management issues, a tangible solution is still required. The patients predominantly come from middle- and low-income populations, which are less educated and clinically more demanding. Moreover, the average per-day footfall is enough to break even a good level of planning and policy. As we are progressing toward the 2030s, government hospitals are also witnessing a rapid transformation to meet the changing demands and emotional needs of the patient population.

The All India Institute of Medical Sciences, Raebareli (AIIMS Raebareli), is a tertiary care center with the objective of providing the best medical services at a subsidized cost and in a reasonable time span. Considering the footfall of over 3,000 patients per day, the administrative ability to cater to the patients’ satisfaction encounters technical challenges. The diversity in education levels and socioeconomic backgrounds of every patient and the plethora of departments running a tertiary-level center increase the difficulty level of managing and providing individualized care. Moreover, the expectation of patients before entering the hospital is always high and comparable to other private centers. The best medical service not only includes high-quality, state-of-the-art consultation and surgery, but also includes timed consultations, timed registration, and easy navigation for patients. In our study, we measured the satisfaction scores of our patients and determined the delay time and time spent at every stage of movement through the hospital. Patient satisfaction is a significant health outcome that is a factor in care quality. The purpose of this study was to evaluate the degree of patient satisfaction with outpatient services and the factors that influence it at a government tertiary care facility. Overall patient satisfaction increased as a result of Lean Six Sigma’s [3] improvisation and revisiting of key crucial indicators, including "Time to Get the OPD Slip", "Time to Payment", and "medical service staff attitudes". Hence, our study is a retrospective analysis of all prospective interventions that were carried out to make hospital services better. The objective of our study was to identify the problems of our patients and factors leading to decreased satisfaction levels so that the hospital administration could act accordingly. Our study may provide an important baseline to which hospital administration policies may refer to guide improvements in similar medical facilities.

## Materials and methods

Between November 2023 and December 2024, a cross-sectional study was conducted in a facility with 400 randomly chosen patients. The formula for the single population percentage was used to get the necessary sample size, assuming a 62% overall patient satisfaction rate, a 95% confidence level, and a 5% margin of error, with an additional 10% allowance for potential nonresponse. This yielded a final sample size of 373 participants. Ethical approval was granted by the Institutional Ethics Committee of Bioethics Cell All India Institute of Medical Sciences, Raebareli, India (2024-1-IMP-EXP-7, 18-05-2024), and informed consent was obtained from all participants prior to data collection. Information was gathered through a pretested, structured interview questionnaire (see Appendix Figures 1-4). Data entry and analysis were performed using SPSS software (IBM, Chicago, Illinois, USA). Prior to statistical analysis, explanatory variables were identified through factor analysis, and bivariate linear regression was conducted for each independent variable in relation to the outcome variable. The significant variables were separately discussed, planned, and intervened. The administrative changes were made in line with the Lean Six-Sigma model. Post-intervention, the same structured interview questionnaire was used again (but for a different subset of patients, considering the outpatient department to be a homogenous environment) to collect data from study participants.

Study design

All interventions were subject to retrospective analysis.

Patient inclusion and study profile

All the questionnaires, along with the grievances and right to information (RTI) complaints received during the financial year 2023-2024, were systematically studied, reviewed, and analyzed to identify recurring issues, assess the effectiveness of service delivery, and understand the concerns raised by patients and their representatives. The analysis covered the nature and frequency of the complaints, the departments and personnel involved, response timelines, and resolution effectiveness.

Exclusion

Partly filled forms (less than 75% filled) or RTIs not pertaining to AIIMS Raebareli were excluded (n = 27 questionnaires that were partly filled were excluded).

Study parameters

The collected information about outpatient department (OPD) service quality served two functions by revealing areas for improvement and demonstrating the need for corrective actions. Globally, health systems are increasingly incorporating patient satisfaction as a key component in evaluations of the quality of medical services. The following documents were analyzed to infer patient satisfaction: (i) A quick response (QR)-based questionnaire of patients at the time of their discharge from the hospital and filled questionnaires of OPD patients, grievances, and RTIs were analyzed for any gaps in our hospital administration services. The suggestion or questionnaire forms were available at the help desk and suggestion box, and an email was already displayed in which patients could randomly fill in their inputs. (ii) Time-motion analyses (time spent at each step from entry to exit of the patient visiting our hospital for outpatient services) were studied objectively and compared. (iii) An anonymous questionnaire, using feedback based on Google Docs (Google LLC, Mountain View, California, USA), was requested from all employees (for intention-to-treat analysis) who had experience as patients in our hospital to evaluate our services, which were segmented into parts from patient entry to exit (for motion analysis).

Dependent variable

The dependent variable was the degree of patient satisfaction with inpatient care.

Independent variables

Sociodemographic Variables

The research analyzed variables that consisted of sex, age, marital status, level of education, occupation, and household income. The assessment checked whether the respondent had a caretaker present with them at the hospital.

Service Utilization-Related Factors

The study collected data on waiting times for services, official visiting hours, service information availability, hospital admission procedures for inpatients, patient privacy measures, confidentiality measures, and signboards in the hospital premises.

Hospital Amenities

This category included factors such as the availability of inpatient accommodation; dietary services offered by the hospital; and the cleanliness of toilets, wards, and beds.

Outcome measurements

Patient Satisfaction (PS)

The 19-item questionnaire assessed patient satisfaction in three main dimensions that included service utilization, interactions between patients and healthcare providers, and facility-related aspects. A five-point Likert scale, with 1 denoting “very dissatisfied” and 5 denoting “very satisfied,” was used to score each item.

Overall Patient Satisfaction (OPS)

Overall satisfaction was measured through a single question in the survey: “How would you rate your overall level of satisfaction with the health services received at this hospital?” The questionnaire used the local language to assist participants in better understanding the questions so they could provide accurate responses.

Data analysis

The data analysis was done using SPSS version 27.0. Descriptive data was presented as frequencies and proportions. Principal component analysis (PCA) was performed to identify the important reasons for poor satisfaction scores among study participants before intervention. For the PCA, the score for each question in the tool was reverse-coded. A Kaiser-Meyer-Olkin (KMO) test and Bartlett’s test were used to identify the adequacy of the sample for PCA. A value above 0.6 in the KMO test was considered significant. A value of 0.8 or more for a variable in each component was considered to identify the responsible variables. An eigenvalue of more than one was considered for the identification of the significant components. Varimax rotation was applied to estimate the final variance of each component. The graphical representation of the significant component was also shown by a scree plot. The pretest and posttest scores for each were compared using an unpaired t-test. A p-value of 0.05 was considered to specify the differences between the comparison groups.

## Results

The study included 373 patients (27 partially completed responses were excluded), resulting in a 90% response rate. Almost 80% of our patients expressed satisfaction with the services they received at the hospital, with the domains related to patient-provider interactions and general facility amenities accounting for 80.4% of the variation in the net overall satisfaction score.

Baseline characteristics of study participants

A total of 261 participants were included in the pre-intervention phase of the study, and 112 participants were included in the post-intervention phase. The baseline characteristics of the study participants are summarized in Table 1. In the pre-test group, males comprised 56.3% (n = 147) and females comprised 43.7% (n = 114).

**Table 1:** Baseline characteristics of study participants during pre-test and post-test interview

		Pre-test (n=261)	Post-test (n=112)
Variable	Category	Frequency (%)	Frequency (%)
Sex	Male	147 (56.3)	57 (50.9)
	Female	114 (43.7)	55 (49.1)
Age (years)	<18	18 (6.9)	6 (5.3)
	18-40	108 (41.4)	58 (51.8)
	>40	135 (51.7)	48 (42.9)
Investigation performed	No	25 (9.6)	7 (6.3)
	Yes	236 (90.4)	105 (93.8)
Follow-up visit	No	67 (25.7)	20 (17.9)
	Yes	194 (74.3)	92 (82.1)
Patients admitted	No	22 (8.4)	2 (1.8)
	Yes	239 (91.6)	110 (98.2)
Patients discharge (pre-test (n = 239) post-test (n = 110))	No	6 (2.5)	1 (0.1)
	Yes	233 (97.5)	109 (99.9)

The post-test group showed a more balanced distribution, with males making up 50.9% (n = 57) and females 49.1% (n = 55) of the participants. Most participants in both the pre-test (51.7%, n = 135) and post-test (51.8%, n = 58) groups were older than 40 years of age. A high percentage of patients underwent an investigation and were admitted in both phases of the study.

Independent variables and patient satisfaction

The three general facility amenities that most affected patient satisfaction were room accommodation, toilet cleanliness, and dietary services. Among these, toilet cleanliness showed the strongest association with the overall satisfaction score. This was further supported by the observation that the highest proportion of “very dissatisfied” responses was related to toilet hygiene, with nearly 40% of participants expressing dissatisfaction with this service. The availability of sufficient waiting space, together with the quality of dietary services, proved to be major factors that influenced customer satisfaction levels. The satisfaction scores showed a strong positive effect derived from pharmacy services under government programs, including Pradhan Mantri Jan Aushadhi and Affordable Medicines and Reliable Implants for Treatment (AMRIT) Yojana (95% CI: 2.1-2.5; p ≤ 0.001). The results of regression analysis showed that patient satisfaction moved from “very dissatisfied” to “very satisfied” as the quality of these services improved. The study revealed an 80.4% satisfaction rate because patients were pleased with the medical care delivered by the doctors and by the conduct of the nursing staff. People were most dissatisfied with the state of toilets and the long lines for getting medicine.

Comparison of pre-test and post-test results

The administrative intervention had a varying impact on patient-reported outcomes (patient satisfaction (PS) and OPS). Overall, the intervention demonstrated a statistically significant positive effect on specific service parameters, indicating improvements in certain areas of the patient experience. The mean scores for various services (comparing the time taken in each step and satisfaction levels), as measured in the pre- and post-intervention phases, are presented in Table 2.

**Table 2:** Gender-wise distribution of mean + SD score of pre-test and post-test among study participants p-value less than 0.05 is considered significant. OPD: outpatient department.

Variable	Male			Female			Total		
	Pre-test Score (Mean ± SD)	Post-test Score (Mean ± SD)	p-Value (t-Test)	Pre-test Score (Mean ± SD)	Post-test Score (Mean ± SD)	p-Value (t-Test)	Pre-test Score (Mean ± SD)	Post-test Score (Mean ± SD)	p-Value (t-Test)
Time to get the OPD slip	4.0 ± 1.0	3.7 ± 1.1	0.414 (-5.5)	4.2 ± 0.9	3.7 ± 1.1	0.018 (-13.59)	4.1 ± 0.1	3.7 ± 1.1	0.011 (-13.99)
Time to meet the doctor	3.6 ± 1.2	3.6 ± 1.1	0.250 (0.00)	3.9 ± 1.0	3.6 ± 1.2	0.193 (-7.42)	3.7 ± 1.1	3.6 ± 1.2	0.952 (-2.37)
Time to payment	4.5 ± 0.8	4.3 ± 0.9	0.608 (-4.53)	4.5 ± 0.8	4.1 ± 0.9	0.043 (-12.83)	4.5 ± 0.8	4.2 ± 0.9	0.213 (-9.62)
Time to get the report	4.1 ± 1.1	3.5 ± 1.4	0.060 (-13.01)	4.0 ± 1.3	3.5 ± 1.5	0.585 (-9.73)	4.1 ± 1.1	3.5 ± 1.5	0.100 (-12.46)
Time to follow-up visit	3.5 ± 1.6	3.3 ± 1.6	0.284 (-3.41)	3.7 ± 1.6	3.4 ± 1.6	0.226 (-5.12)	3.6 ± 1.6	3.3 ± 1.2	0.234 (-5.79)
Time to admit	4.5 ± 0.8	4.2 ± 1.1	0.084 (-8.52)	4.5 ± 0.9	4.2 ± 1.1	0.219 (-8.15)	4.5 ± 0.9	4.2 ± 1.1	0.040 (-8.15)
Time to discharge	4.2 ± 1.0	3.9 ± 1.2	0.074 (-7.42)	4.2 ± 1.0	4.0 ± 1.1	0.691 (-5.20)	4.2 ± 1.0	4.0 ± 1.1	0.195 (-5.20)
The cleanliness of the OPD	4.1 ± 0.8	4.2 ± 0.8	0.229 (+3.41)	4.3 ± 0.7	3.9 ± 0.9	0.006 (-10.16)	4.2 ± 0.7	4.0 ± 0.8	0.203 (-7.27)
Behavior of the doctor, nursing officer, and other hospital staff	4.2 ± 0.8	4.2 ± 0.9	0.823 (0.0)	4.4 ± 0.7	4.1 ± 0.8	0.011 (-10.90)	4.3 ± 0.7	4.1 ± 0.8	0.206 (-7.27)
The communication with your doctor	4.2 ± 0.8	4.3 ± 0.7	0.357 (3.63)	4.3 ± 0.7	4.1 ± 0.7	0.341 (-7.80)	4.3 ± 0.7	4.2 ± 0.7	0.846 (3.90)
The quality of the care you received	4.2 ± 0.8	4.2 ± 0.7	0.155 (0.0)	4.3 ± 0.7	4.2 ± 0.7	0.505 (-3.90)	4.2 ± 0.8	4.2 ± 0.7	0.456 (-0.00)
The privacy of the physician's consultation room and the examination room	4.3 ± 0.8	4.2 ± 0.8	0.465 (-3.41)	4.3 ± 0.7	4.0 ± 0.7	0.010 (-11.71)	4.3 ± 0.7	4.1 ± 0.8	0.520 (-7.27)

After the intervention, a statistically significant improvement was observed in the “Time to Get the OPD Slip” for the total study population. The mean score improved from 4.1 ± 0.1 to 3.7 ± 1.1 (p = 0.011). This improvement was particularly significant among female participants, whose mean scores for this variable improved from 4.2 ± 0.9 to 3.7 ± 1.1 (p = 0.018). We would like to highlight the fact that administrative intervention was performed to make a separate queue system for males and females, especially for the abled and senior citizens. For the domain, “Time to Payment,” a significant improvement was noted specifically among female participants, with the mean score improving from 4.5 ± 0.8 to 4.1 ± 0.9 (p = 0.043). However, the change in the total population (pre and post) was not statistically significant (p = 0.213). Other service time variables, including “Time to Meet the Doctor” and “Time to Get the Report,” did not show statistically significant changes in the overall study population. This may be because an efficient and automated reception hospital management information system (HMIS) pushed a larger number of patients to doctor’s chambers and thereby increased the waiting time outside doctors’ offices.

Principal component analysis of patient responses

The suitability of the data for PCA was confirmed by a KMO measure of 0.868, indicating a meritorious sampling adequacy and a statistically significant Bartlett’s Test of Sphericity (p = 0.000). The analysis extracted five principal components with eigenvalues greater than 1, collectively explaining 68.8% of the total variance in patient responses (Tables 3, 4).

**Table 3:** Principal component analysis of patient responses in our study KMO: Kaiser-Meyer-Olkin.

KMO and Bartlett's Test		
Kaiser-Meyer-Olkin measure of sampling adequacy		0.868
Bartlett's Test of Sphericity	Approx. Chi-square	898.579
	df	78
	Sig.	0.000

**Table 4:** Cumulative variance accounted for by PCA of patient response data Total variance explained. PCA: principal component analysis.

Component	Initial Eigenvalues			Extraction Sums of Squared Loadings			Rotation Sums of Squared Loadings		
	Total	% of Variance	Cumulative %	Total	% of Variance	Cumulative %	Total	% of Variance	Cumulative %
1	5.039	38.760	38.760	5.039	38.760	38.760	3.903	30.024	30.024
2	1.803	13.871	52.631	1.803	13.871	52.631	1.866	14.355	44.379
3	1.103	8.484	61.115	1.103	8.484	61.115	1.716	13.197	57.576
4	1.000	7.690	68.806	1.000	7.690	68.806	1.220	9.388	66.964
5	0.777	5.978	74.784	0.777	5.978	74.784	1.017	7.820	74.784

The rotated component matrix (Table 5) revealed the factor loadings for each variable, allowing for the interpretation and naming of each component.

**Table 5:** Rotated component matrix of patient responses in our study ^a^Extraction method: principal component analysis, and rotation method: varimax with Kaiser normalization. Rotation converged in five iterations. OPD: outpatient department, IPD: inpatient department.

Rotated Component Matrix^a^					
	Component				
	1	2	3	4	5
The quality of the care you received	0.888	0.096	0.112	0.090	-0.078
Behavior of the doctor, nursing officer, and other hospital staff	0.885	0.127	0.067	0.087	-0.022
The communication with your doctor	0.864	0.146	0.191	0.119	0.029
The privacy of the physician's consultation room and the examination room	0.823	0.122	0.105	0.100	0.095
The cleanliness of the OPD and IPD	0.799	0.041	0.193	0.013	0.052
Time to payment	0.097	0.811	0.213	-0.182	0.038
Time to admit	0.184	0.733	-0.122	0.311	0.130
Time to get report radiology	0.063	0.553	0.222	0.502	-0.019
Pathology	0.095	-0.055	0.864	0.095	0.092
Time to meet doctor	0.313	0.261	0.628	0.191	0.014
Time to get OPD slip Online ( ) Offline ( )	0.288	0.469	0.567	0.109	0.001
Time to follow-up visit (revisit to report)	0.180	0.044	0.186	0.853	0.135
Time to discharge	0.023	0.099	0.088	0.115	0.976

Quality of Interaction

This component accounted for 30.024% of the variance and was strongly loaded by variables related to the quality of care received (0.888), the behavior of staff (0.885), communication with the doctor (0.864), and patient privacy (0.823). This component highlights the importance of interpersonal and qualitative aspects of care. Our hospital has separate rooms for individual consultations, and the policy of consulting one at a time exists. Although the waiting time was longer, overall satisfaction remained high because after the consultation, patients felt a sense of belonging and appreciated the quality treatment.

Time to Access Services

This factor explained 14.355% of the variance and was defined by variables such as “Time to Payment” (0.811) and “Time to Admit” (0.733). This component reflects the patient’s experience with the administrative and logistical efficiency of the hospital. We would like to highlight that patients and even caregivers accept the need to wait for their doctor but feel “unsatisfied” in the queue at reception. Henceforth, administrative policies should focus on these petty issues to improve overall quality care.

Clinical Efficiency

This component, which explained 13.197% of the variance, had high loadings on “Pathology Report Time” (0.864) and “Time to Meet the Doctor” (0.628). This suggests a day-to-day phenomenon in every government hospital. After the patient has consulted with their doctors, a few investigations are usually advised. Although the staff (and hence staff behavior) remains largely the same, patients and their caregivers often lose patience and develop feelings of dissatisfaction and annoyance.

Radiology and Cleanliness

This component accounted for 9.388% of the variance and was primarily driven by “Time to Get the Radiology Report” (0.502) and “Cleanliness of the OPD and IPD” (0.193).

Follow-up and Discharge

The final component explained 4.276% of the variance, with the strongest loading on “Time to Follow-Up and Revisit” (0.892). Here also, the staff (and hence staff behavior) remained much the same, but patients and their caregivers lost patience and developed feelings of dissatisfaction and annoyance. Hence, policymakers should focus on revisits, follow-ups, and revisits to reception counters for either billing, reports, or laboratory services. A robust follow-up Tele-OPD service could provide solutions to all the above issues. We have developed the Tele-OPD services and have drafted the relevant administrative policy and hope to provide data in a future article.

Planning and administering interventions: the Lean Six Sigma model

The implementation of the Lean Six Sigma [3] methodology provided the foundational framework for this study, guiding both the diagnostic phase and the subsequent interventions. As a robust process improvement model, it enabled us to move beyond simple observations to a data-driven, systematic approach. The Define and Measure phases involved identifying critical patient-reported issues and collecting quantitative data through questionnaires to establish baseline metrics (pre-test data). The Analyze phase, using statistical tools such as PCA, allowed us to pinpoint the root causes of dissatisfaction, including the qualitative aspects of “Quality of Interaction” and the inefficiency of specific workflows. This informed the Improve phase, in which targeted interventions were designed to eliminate process waste (Lean) and reduce variability (Six Sigma). Finally, the Control phase, reflected in the post-test data collection, was used to statistically validate the effectiveness of the interventions. This rigorous methodology ensured that the improvements were not anecdotal but were statistically significant and directly correlated with the implemented changes, as demonstrated by the positive shifts in key patient satisfaction metrics.

Our strategic interventions for quality improvement

The following interventions were strategically implemented within a quality improvement framework, such as Lean Six Sigma, to address identified deficiencies in patient satisfaction and operational efficiency.

Digital and Workflow Optimization

The core of this intervention set was the application of digital technology and process reengineering to streamline patient flow and reduce administrative burden.

Digitalization of patient entry: The implementation of a digital registration platform via the Ayushman Bharat Digital Health Mission served to standardize and accelerate the initial patient intake process. This is consistent with studies on digital health integration, which demonstrate its efficacy in reducing data entry errors and in improving administrative efficiency (Smith and Keselman, 2020) [4].

Stratified queue management:The introduction of a queue management system with segmented tokens for different patient demographics directly addressed patient flow theory by reducing cognitive friction and perceived wait times. This strategy minimizes confusion and allows more efficient resource allocation, a key tenet of lean management, to eliminate waste in the form of waiting.

Integration of digital systems: The deployment of a unified digital billing system and the extension of the HMIS to outpatient departments created a seamless digital ecosystem. This move from disparate, manual systems to integrated digital records is a foundational step in modernizing healthcare operations and ensuring data integrity and accessibility (Intorne et al., 2023) [5].

Human and Environmental Reengineering

These interventions focused on the physical environment and the human-centric aspects of service delivery. PCA identified as critical to patient satisfaction.

Environmental service optimization: The reengineering of housekeeping staff shifts to start earlier is a direct application of process optimization to improve the physical environment. A clean and well-maintained facility is a key indicator of quality for patients and contributes to a positive perception of care, as outlined in patient-centered care models.

Workflow streamlining: The reengineering of the workflow to allow investigations to be raised directly in the OPD and to permit the establishment of separate reception counters for IPD, Emergency, and OPD patients is a direct application of lean principles to eliminate redundant steps and reduce patient wait times. This reduces the number of handoffs and queues, thereby improving logistical efficiency. Accordingly, we would like to highlight the importance of R-1. Initially, we faced resistance from all doctors for changing the pattern (R-1); but later on, doctors realized the importance of administrative change and accepted the workflow.

Service Quality and Personnel Development

This set of interventions addressed the human element of healthcare, which our study found to be the most significant factor in overall satisfaction.

Patient-centric staff training: The provision of regular soft skills training to staff is a proactive measure to cultivate a patient-centric culture. The literature consistently demonstrates that effective communication, empathy, and professional behavior from hospital staff are of paramount importance for building patient trust and directly correlate with higher satisfaction scores (Chen et al., 2024) [6]. Regular training on handwashing, three-bucket system cleanings, spill management, and on-site segregation of biomedical waste was conducted.

Performance-based service models: The shift from a manpower outsourcing to a service-quality outsourcing model represents an innovative approach for aligning operational incentives with quality outcomes. By tying compensation and contracts to service quality metrics, this model encourages a commitment to patient satisfaction beyond basic task completion. This moves the focus from quantity to quality, a central theme in Six Sigma methodology. Previously, our housekeeping was managed by manpower contracts. Later, we changed the mode of work to service contracts and to quality outcomes (as in airports).

## Discussion

According to the results of our study, 80.4% of patients were satisfied overall. It is pertinent to mention here that healthcare service is different than other service sectors like banking, railways, engineering, and consultancy because of intangible and emotional factors. It should be noted that the patients visiting the hospital are not generally in a sound physical or mental state, which may affect their ability to communicate or understand medical instructions clearly. Every patient believes that they should be a priority and that they should be attended to first. Thus, in a continuously evolving healthcare facility, the challenge to improve the satisfaction levels of patients remains a critical area for any hospital administrator. The problem is even worse in government tertiary care institutes, where large numbers of patients are referred in terminally ill or critical conditions. The patient treats the government hospital as the last ray of hope, and therefore, it becomes the responsibility of hospital administrators to improvise and improve upon their services to meet the growing public demand. Every patient has expectations of the healthcare provider, and a positive medical consultation is one of the important elements of that expectation. Apart from the doctor’s behavior, patients usually expect timely delivery of health services, a clean and comfortable environment, and an empathetic staff. In our study, we have focused on the timed delivery of healthcare in our facility and on the behavior of staff toward patients, considering these two as modifiable factors.

Patient satisfaction has been defined as a patient’s reaction to several aspects of their service experience [7]. It is important to measure patient satisfaction to gain valuable and unique insights into daily hospital care and quality. Accordingly, the evaluation of a patient’s satisfaction is a relative term, depending upon socioeconomic, cultural, and educational factors along with the type of disease, age of the patients, and their experiences in other hospitals. The patient’s response largely depends upon their individual expectations and evaluations of health services. In our study, we measured not just the response but also intervened according to the response. This pre- and post-intervention study from the management side studied the improvement in the patients’ responses. In this study, we attempted to analyze the statistically significant factors (time, behavior, and environment) affecting the satisfaction of patients visiting a government tertiary care hospital. Ferreira et al. conducted a similar study (meta-analysis) to analyze the factors underlying patient satisfaction [7]. The study was not only helpful in improving satisfaction but also helped the managers understand the psychology of patients and further develop an organizational policy. From the larger domains of the Donabedian framework, we removed some essential components and constructed a questionnaire in a language that the patients and their attendants could easily understand and implement. The questionnaires were filled out at the time of exit after the patient had used the services of our hospital.

Various attributes have been linked to patient’s satisfaction in the literature, as follows: (a) Patient-related attributes included demographics such as age, gender, education, marital status, socioeconomic position, race, religion, geography, frequency of visits, duration of stay, health, personality, and expectations. (b) Management-related attributes included physical surroundings, availability, accessibility, financial aspects, organizational traits, ambiance, cleanliness, temperature, lighting, food, comfort, equipment, facilities, and parking, as well as the locations, waiting times, admission and discharge procedures, the effort required to schedule an appointment, payment flexibility, insurance status, and insurance coverage. (c) Staff-related attributes included technical abilities, interpersonal care, continuity of care, nursing care, friendliness, concern, empathy, compassion, civility, and respect, as well as the quality of care and the quantity of physicians, nurses, facilities, and equipment. Courtesy, friendliness, kindness, approachability, respect, responsiveness, attentiveness, and care are among the positive attitudes of the provider.

In the systematic review by Ferreira et al. [7], the three most important factors affecting patients’ satisfaction were waiting time, nursing care, and doctors’ characteristics. Analyzing the factors more closely, we found that doctors’ characteristics (empathy, consulting style, and the doctor-patient relationship) remained the most important attributes in a public hospital. It seems that patients were well aware of the fact that waiting time would be longer in a public hospital. Similar results were observed in this study, wherein, contrary to our belief, the only significant factor was the doctor’s behavior and humane response toward the care of their patients. The waiting time was a determinant of healthcare dissatisfaction, regardless of the inpatient stage. Waiting time is clearly an obstacle to access, but management may change the workflow to improve the situation, so this factor is modifiable.

Our quality improvement process became more rigorous after we adopted Lean Six Sigma as the framework for planning and administering interventions. The model enabled us to progress through the diagnostic, intervention, and validation stages one step at a time. The Define and Measure phases used questionnaires and baseline (pre-test) data collection to identify critical concerns expressed by patients. During the Analyze phase, we employed PCA to statistically explore the underlying constructs of dissatisfaction. The PCA showed that Quality of Interaction (doctor-patient behavior and empathy) and Workflow Inefficiencies (delays in registration, billing, and report turnaround) were the two major latent domains that most strongly influenced the patient experience. The research findings supported our initial descriptive analysis by showing statistical evidence that supported the selection of changeable risk factors. The Improve phase implemented targeted interventions based on these insights, which included digitalizing patient entry and billing systems, stratifying queue management, optimizing housekeeping schedules, opening all outpatient department clinics on a timely basis, and enhancing staff soft-skills training. The Control phase confirmed that post-intervention data collection showed statistically significant improvements that resulted in measurable patient satisfaction gains, primarily in interpersonal behavior and timely service delivery. The structured approach allowed us to track changes that resulted from our interventions so we could prove Six Sigma’s value in hospital administration.

Because the number of corporate hospitals with state-of-the-art medical facilities and equipment is growing, a paradigm shift toward quality care improvement in public hospitals is needed. These kinds of studies are mostly found in Western literature. Countries like Japan and the U.S. focus on total quality management and measure their healthcare systems in terms of the satisfaction levels of the patients being treated. Given the setting of a tertiary care institute that must validate the faith of the general population of the country, our study stands as an important turning point for future planning and policy making. In an Italian study by Alibrandi et al., the authors highlighted that satisfaction not only depends upon the effectiveness of the treatments and the physicians’ competence for determining patient satisfaction but also upon the overall workflow and policy of hospital management [8]. The authors highlighted an important limitation of these “satisfaction studies” that exists in our study as well. Patient satisfaction measurements capture several components of “happiness” that are easily impacted by circumstances unrelated to care, which makes the patient input unreliable [8,9]. The authors developed a questionnaire with a basic methodology very similar to this study’s. Alibrandi et al.’s questionnaire incorporated (a) pre-booking, which includes the ease of booking, getting to the hospital, and ticket payment; (b) issues involving movement at the hospital, which includes arriving at the hospital (parking and building access challenges) and having a medical consultation (waiting times, comfort, ambulatory hygiene, and the conduct of medical and nursing personnel); and (c) the patient’s experience following the visit: in particular, the information provided about the treatment to be followed, the phone numbers of those to contact in case of emergency, and the ease with which the medical findings could be obtained [8]. In our questionnaire, we obtained feedback from the patients about the time required to take various steps from registration to consultation, investigations, and obtaining reports. Additionally, satisfaction was also checked in the context of the time taken in the above steps. Unlike Alibrandi’s study, we analyzed significant factors, intervened administratively to change the workflow, and measured the change in satisfaction level cross-sectionally. Among the factors studied, the behavior of nurses and doctors, cleanliness, and the ease of booking and parking were statistically significant factors. In our study, we tried to intervene in all these factors to check for improvement, but only one factor was found to be significant throughout: the behavior of the doctors. Liu et al. found that “patient’s characteristics” are also significant in determining outcomes [10]. Higher-educated patients expressed greater satisfaction with the administrative procedure, according to the author’s findings. The survey revealed different satisfaction levels based on age group and sex, as well as residential location. Older patients, together with those who reported poor health status, showed lower satisfaction regarding the hospital environment [10]. The determinants of satisfaction varied notably between public and private hospitals because of differences in work culture. Public hospitals, as nonprofit institutions, cater to a large patient load on a daily basis, whereas private facilities frequently assess patient satisfaction and use the results as performance indicators [10].

A review by Li et al. [11] of outpatient satisfaction found that patients were most pleased with doctors and nurses but showed the least satisfaction with hospital hygiene and outpatient procedures, especially long waiting times. The research highlighted the requirement to tackle changeable elements, which included service provider attitudes among pre-diagnosis nurses and registration staff and at pharmacy counters [11]. The authors proposed creating policies to decrease waiting periods, particularly for patients with standard medical needs [11].

In the present study, further steps were taken to examine system improvements and evaluate policy implementation effects. Consistent with other findings, satisfaction with doctors and nurses showed a strong association with overall satisfaction [11]. The improvement of doctor-patient relationships continues to be essential, but maintaining this in tertiary care hospitals becomes difficult because faculty members usually have too much work. Li et al. [11] showed that people with higher incomes and higher educational levels reported lower satisfaction with outpatient care, which matched our findings. This outcome may be the reason for the overall high satisfaction levels in our patients. Our hospital caters to a low socioeconomic population, and patients are usually from less educational backgrounds, so doctors’ empathy translates into higher satisfaction. Additionally, research indicates that older patients are typically less happy with hospital information experiences, including online registration and contemporary payment options. Therefore, older patients must receive some technological aid to help them adjust to new advances [11]. Similar results were replicated by a study in Ethiopia (a low socioeconomic country) [12]. Our findings will undoubtedly have a big impact on how healthcare practitioners in poorer nations approach their work. Additionally, Asamrew et al. discovered that the net overall satisfaction level of the patients was strongly predicted by the inpatient pharmacy service [12]. We did not evaluate this factor because we do not have an inpatient pharmacy, but we believe that this may be an important predictor. A study [13] identified six objectives for a quality healthcare system: (a) safety, (b) equity, (c) evidence-based practice, (d) timeliness, (e) efficiency, and (f) patient-centeredness, with the latter three aspects directly impacting patient satisfaction. In our study, the time factor was not statistically significant, but the data supported a similar inference. In another Indian study by Nilakantam et al. [14], 84% of patients were asked to respond to a survey, and the patient satisfaction rate for the clinical care provided by the doctor was 97%. Factors like “explanation of the disease by the doctor,” “necessity of the investigation suggested,” “opportunity given to talk” about their illness, and “listening skills of the doctor” were significant in Nilakantam’s study [14]. After analyzing the literature and our results, we found that the doctor’s behavior has the greatest effect on patient satisfaction. A successful appointment with a reputable and trustworthy consultant will thus have positive benefits regardless of any additional therapy provided because a positive doctor-patient connection is therapeutic in and of itself [14]. Other studies from India showed varied satisfaction levels from 70% to 95% [15-19].

Limitations of this study

First, we believe that the post-intervention population was different from the pre-intervention sample, so a selection bias exists; however, in timed cross-sectional studies, administrative interventions are not possible on the same day. Second, we did not group the patients by educational status; otherwise, satisfaction across different educational strata would have given more insights. We had planned to keep this parameter in our methodology, but we found that the population was homogenous, and saturation was achieved too early regarding this question. This may be because most patients belonged to the same socioeconomic and educational background. The evaluation of the physicians’ competencies is influenced by potential bias: the patient utilizing the healthcare service will invariably express satisfaction because they are the individuals selecting the polyclinic hospital, setting the appointment, and awaiting the consultation or clinical assessment. Data were gathered and utilized from a convenience sample, which was limited to patients who were contacted and provided their consent, and therefore were not representative of the entire patient population. This is because the patients had to provide their agreement to be contacted, which meant that there was an inevitable self-selection of the sample during sample building.

## Conclusions

The study revealed that overall satisfaction with outpatient services at this tertiary care hospital was quite high (80%), which is encouraging; however, there are still opportunities for improvement. The satisfaction level in this study was somewhat lower than in other studies, especially those conducted in corporate-managed hospitals. The combination of better patient-provider communication with improved facility amenities should result in significant growth in patient satisfaction. Equally important is the improvement of health literacy among service providers and the establishment of a systematic approach to routinely measure patient satisfaction. Healthcare workers require frequent customized on-the-job training to provide patient-centered, high-quality care. Our findings demonstrate that medical service staff, including nurses, registration and pre-diagnosis personnel, and pharmacy staff, need to enhance their service attitudes. To reduce waiting times, adopting policies that integrate technology and automation for effective crowd management could be a valuable step forward.

## Figures and Tables

**Figure 1:**
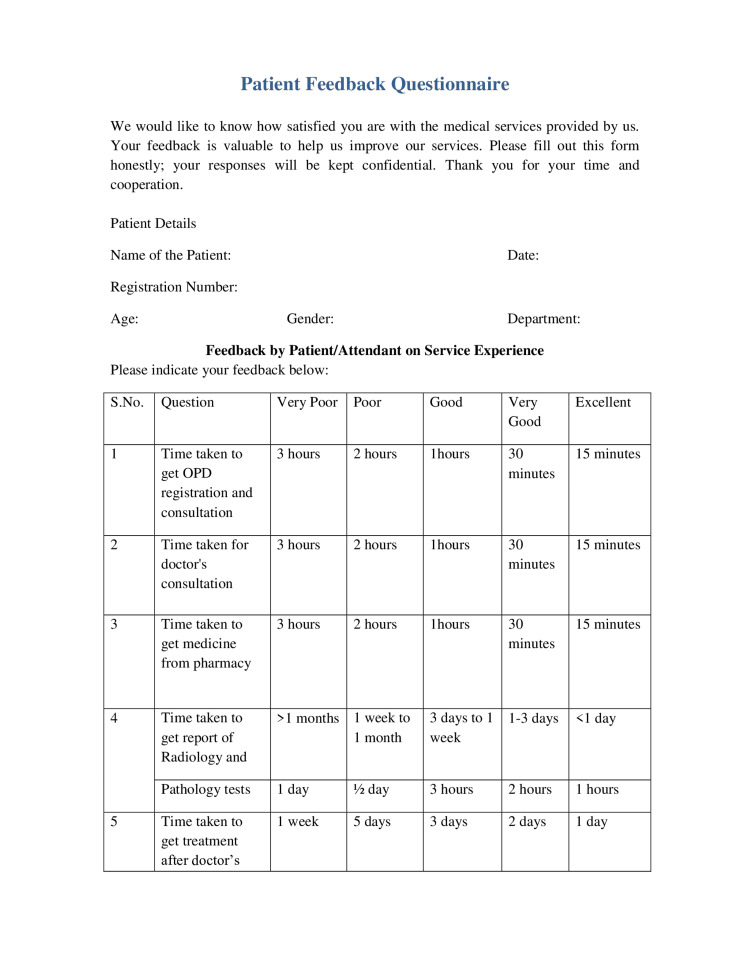
Sample of questionnaire/proforma translated into English (page 1) OPD: outpatient department.

**Figure 2:**
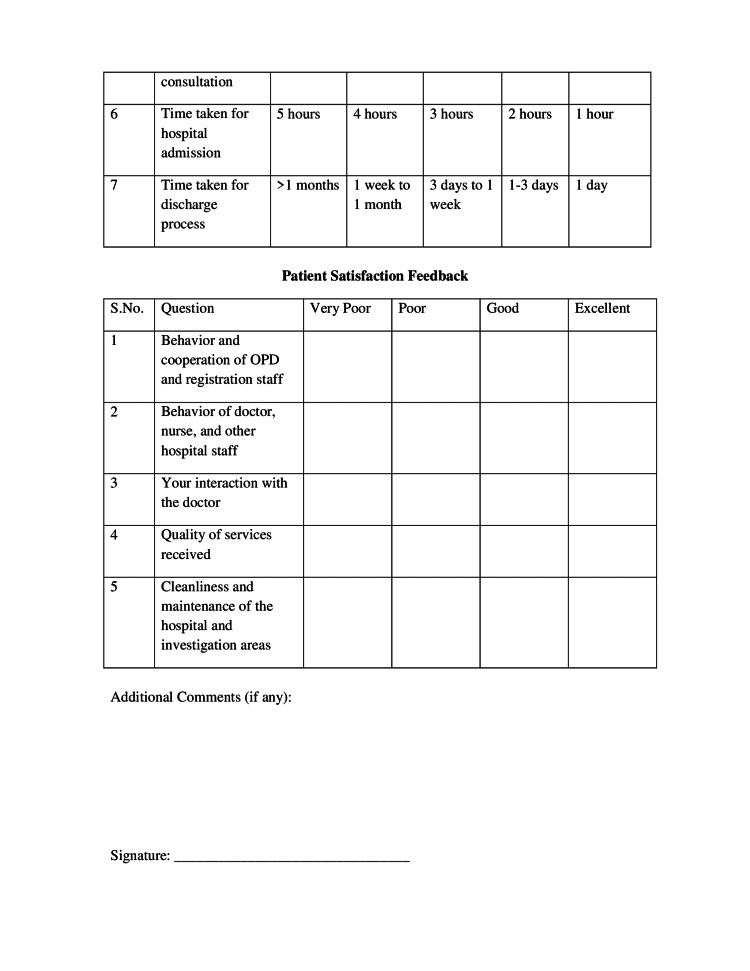
Sample of questionnaire/proforma translated into English (page 2) OPD: outpatient department.

**Figure 3:**
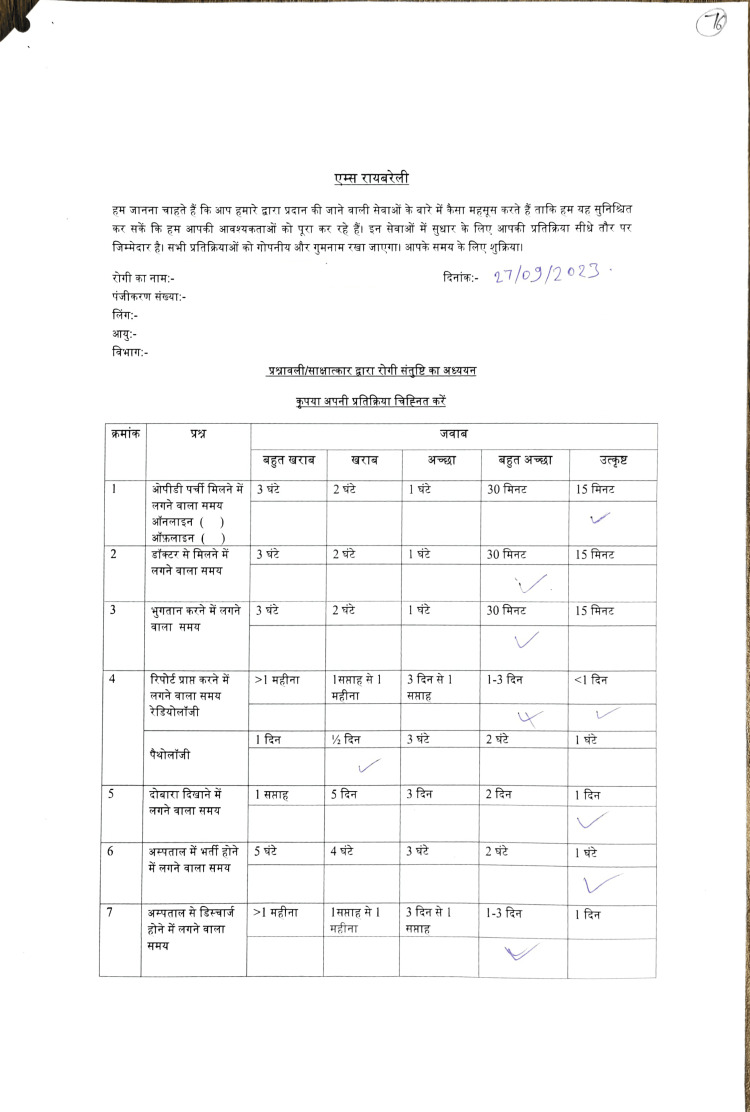
Sample of questionnaire/proforma filled by patient (page 1) Name and personal details of the patients or their attenders have been anonymized.

**Figure 4:**
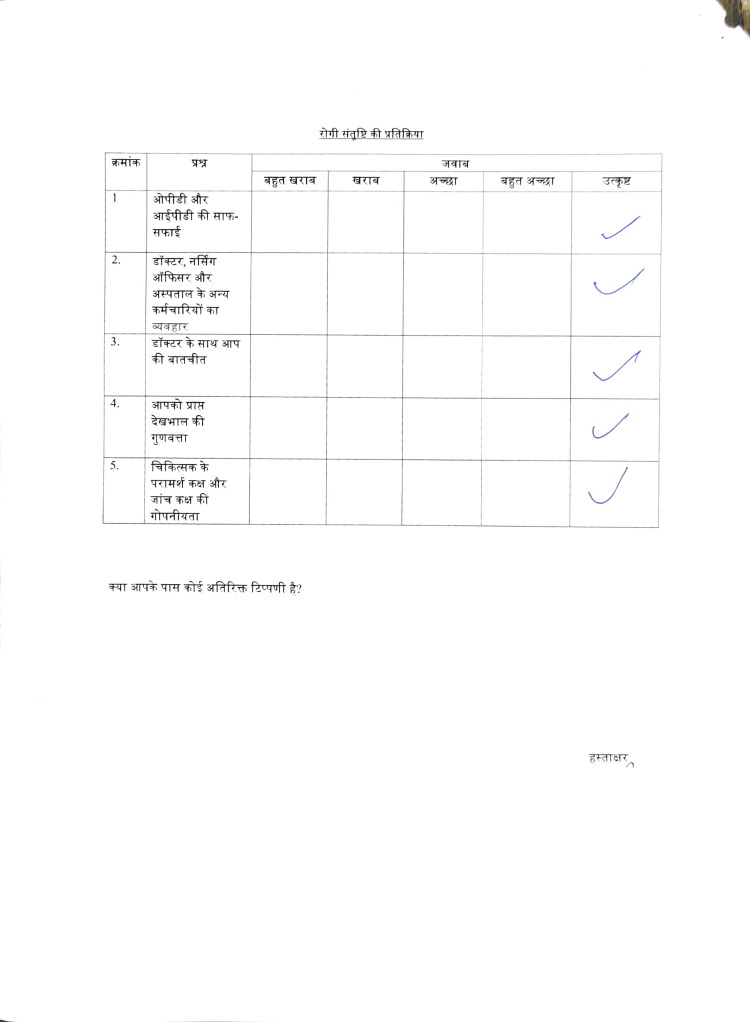
Sample of questionnaire/proforma filled by patient (page 2) Name and personal details of the patients and their representative have been anonymized.
